# Practice Doesn’t Always Make Perfect: A Qualitative Study Explaining Why a Trial of an Educational Toolkit Did Not Improve Quality of Care

**DOI:** 10.1371/journal.pone.0167878

**Published:** 2016-12-28

**Authors:** Janet A. Parsons, Catherine H. Y. Yu, Natalie A. Baker, Muhammad M. Mamdani, Onil Bhattacharyya, Merrick Zwarenstein, Baiju R. Shah

**Affiliations:** 1 Applied Health Research Centre, Li Ka Shing Knowledge Institute, St. Michael’s Hospital, Toronto, Ontario, Canada; 2 Department of Physical Therapy, University of Toronto, Toronto, Ontario, Canada; 3 Division of Endocrinology and Metabolism, Department of Medicine, and Li Ka Shing Knowledge Institute, St. Michael’s Hospital, Toronto, Ontario, Canada; 4 Department of Medicine, University of Toronto, Toronto, Ontario, Canada; 5 Dalla Lana School of Public Health, University of Toronto, Toronto, Ontario, Canada; 6 Li Ka Shing Centre for Healthcare Analytics Research and Training, Li Ka Shing Knowledge Institute, St. Michael’s Hospital, Toronto, Ontario, Canada; 7 Leslie Dan Faculty of Pharmacy, University of Toronto, Toronto, Ontario, Canada; 8 Institute of Health Policy Management and Evaluation, University of Toronto, Toronto, Ontario, Canada; 9 Women’s College Hospital Research Institute, Women’s College Hospital, Toronto, Ontario, Canada; 10 Department of Family and Community Medicine, University of Toronto, Toronto, Ontario, Canada; 11 Centre for Studies in Family Medicine, Department of Family Medicine, Western University, London, Ontario, Canada; 12 Institute for Clinical Evaluative Sciences, Toronto, Ontario, Canada; 13 Sunnybrook Health Sciences Centre, Toronto, Ontario, Canada; University of Stirling, UNITED KINGDOM

## Abstract

**Background:**

Diabetes is a chronic disease commonly managed by family physicians, with the most prevalent complication being cardiovascular disease (CVD). Clinical practice guidelines have been developed to support clinicians in the care of diabetic patients. We conducted a pragmatic cluster randomized controlled trial (RCT) of a printed educational toolkit aimed at improving CVD management in diabetes in primary care, and found no effect, and indeed, the possibility of some harm. We conducted a qualitative evaluation to study the strategy for guideline implementation employed in this trial, and to understand its effects. This paper focuses solely on the qualitative findings, as the RCT’s quantitative results have already been reported elsewhere.

**Methods and Findings:**

All family practices in the province of Ontario had been randomized to receive the educational toolkit by mail, in either the summer of 2009 (intervention arm) or the spring of 2010 (control arm).A subset of 80 family physicians (representing approximately 10% of the practices randomized and approached, with records on 1,592 randomly selected patients with diabetes at high risk for CVD) then took part in a chart audit and reflective feedback exercise related to their own practice in comparison to the guideline recommendations. They were asked to complete two forms (one pre- and one post-audit) in order to understand their awareness of the guidelines pre-trial, their expectations regarding their individual performance pre-audit, and their reflections on their audit results. In addition, individual interviews with thirteen other family physicians were conducted. Textual data from interview transcripts and written commentary from the pre- and post-audit forms underwent qualitative descriptive analysis to identify common themes and patterns. Analysis revealed four main themes: *impressions of the toolkit*, *awareness was not the issue*, *‘it’s not me it’s my patients’*, and *chart audit as a more effective intervention than the toolkit*. Participants saw neither the toolkit content nor its dissemination strategy to be effective, indicating they perceived themselves to be aware of the guidelines pre-trial. However, their accounts also indicated that they may be struggling to prioritize CVD management in the midst of competing demands for their attention. Upon receiving their chart audit results, many participants expressed surprise that they had not performed better. They reported that the audit results would be an important motivator for behaviour change.

**Conclusions:**

The qualitative findings outlined in this paper offer important insights into why the intervention was not effective. They also demonstrate that physicians have unperceived needs relative to CVD management and that the chart audit served to identify shortcomings in their practice of which they had been hitherto unaware. The findings also indicate that new methods of intervention development and implementation should be explored. This is important given the high prevalence of diabetes worldwide; appropriate CVD management is critical to addressing the morbidity and mortality associated with the disease.

## Introduction

Diabetes is a chronic disease commonly encountered by family physicians, and its prevalence is on the rise[1, 2]. It carries with it significant morbidity and mortality as well as substantial economic costs[3, 4]. Diabetes care is complex and entails screening for multiple risk factors and managing a range of complications resulting from the disease [5]. The most prevalent complication is cardiovascular disease (CVD), which accounts for more than half the mortality among diabetic patients[4, 5]. While clinical practice guidelines have been developed to support clinicians [6–8], unfortunately the care patients actually receive in practice frequently falls short of the guideline recommendations [2, 9–13].

Although the development of clinical practice guidelines has become increasingly popular (and in a wide variety of practice contexts), their promise of improving patient care and disease management frequently goes unrealized. The reasons for this are complex, and guideline uptake can be influenced by features of the practice environment, of the dissemination and implementation strategies, and of the evidence shared [14]. Studies of printed educational materials tied specifically to clinical practice guidelines have shown varying results [5, 15–17]. A Cochrane systematic review revealed that printed educational materials (PEMs) may only offer slight improvements in professional practice outcomes when compared to no intervention; there were insufficient results to offer any conclusion regarding their impact on patient outcomes [16]. A large pragmatic cluster RCT of PEMs aimed at family physicians failed to improve retinal screening uptake in patients with diabetes [17]. Guideline uptake is clearly complex and issues of development, messaging and format all play a role [18].

In December 2008, the Canadian Diabetes Association (CDA) updated their national clinical practice guidelines for diabetes. In an effort to support uptake of these guidelines, the CDA created an educational toolkit to be mailed to family physicians across the country with the aim of improving the management of cardiovascular risk factors and outcomes among people with diabetes [6]. A pragmatic cluster randomized controlled trial (RCT) was conducted to evaluate the effectiveness of this toolkit. A pragmatic approach was adopted, because it was recognized that more traditional explanatory trials aim to determine whether an intervention will work under ‘ideal’ conditions, while pragmatic designs seek to determine effectiveness under “usual-care, real world” conditions, making them better suited for research in primary care settings [19]. A detailed description of the intervention and study design of the trial has previously been published [20], and the quantitative results of the RCT have been reported previously elsewhere [5]. The quantitative results from the RCT indicated that the toolkit implementation did not improve quality of care or cardiovascular outcomes in the study sample, leading to the conclusion that PEMs were not effective [5]. We conducted a qualitative process evaluation [21] in order to study the strategy for guideline implementation employed in this RCT, and to understand why it was not effective. This paper focuses solely on the qualitative findings, reporting important data focused on physicians’ perspectives regarding the trial which was not included in the prior publication.

### Background information on the intervention and RCT design and results

It is important to contextualize the qualitative study within the broader investigation and to outline the dissemination strategy employed. While it has been described in detail elsewhere [5,20], we outline it briefly here, emphasizing pertinent details.

The CVD toolkit was created for the CDA by a team of clinical experts, including endocrinologists, family physicians, and other health care professionals, as well as guidance from clinicians with expertise in knowledge translation [5]. The 2008 guidelines contained a (new) emphasis on prioritizing CVD risk factor screening and management [6]. Packaged in a brightly coloured box with CDA (and pharmaceutical company) branding, it contained: an introductory letter from the Chair of the practice guidelines’ Dissemination and Implementation Committee; an eight-page summary of selected sections of the practice guidelines targeted towards family physicians; a four-page summary of key guideline elements related to CVD risk; a small double-sided laminated card with a simplified algorithm for cardiovascular risk assessment, vascular protection strategies and screening for CVD; and a pad of tear-off sheets for patients printed with a cardiovascular risk self-assessment tool and a list of recommended risk reduction strategies [20].

The implicit theory underlying the intervention strategy was that the full set of written guidelines (201 pages) was too long and complex for rapid integration into clinical practice. The toolkit simplified the information, tailored it to the practice needs of family physicians, and offered actionable recommendations for patient care. The underlying theory of change [22] was that the introduction of the toolkit would influence physicians’ practice behaviours and translate into improved outcomes for their patients.

The toolkit was mailed to most family physicians in Canada, but in the province of Ontario family practices were randomized to either the intervention or control group (1:1 ratio). All Ontario family practices (n = 4,700) were randomized to receive the toolkit by mail, in either the summer of 2009 (intervention arm) or the spring of 2010 (control arm)[5, 20]. This staggered mail out meant that those who did not receive the first mail out acted as controls. Physicians were blinded to the fact that they were part of a randomized trial.

A total of 933,789 of Ontario residents aged ≥ 40 years diagnosed with diabetes were randomized, and studied using population-level healthcare administrative databases [5]. Additional clinical outcome data were collected from randomly selected practices from each of the intervention and control arms (40 of each), with one physician randomly selected from within each practice. A random sample of 1,592 patients who were considered at high risk for CVD were recruited from each of these 80 physicians’ practices [5]. The primary outcome for the administrative data investigation was death or non-fatal myocardial infarction, which occurred in 2.5% of patients in the intervention and control group respectively (p = 0.77) [5]. The primary outcome in the clinical data study was use of a statin (either initiated or ongoing), with 88.1% of patients in the intervention arm and 90.1% in the control arm receiving one (p = 0.26) [5]. The intervention showed no effect on *most* of the pre-specified secondary outcomes—including processes of care, other clinical events and measures of risk factor control [5]. Interestingly, the administrative data investigation revealed statistically significantly worse outcomes for the intervention group on ECGs (38.8% vs. 40.2%, OR = 0.96, p = 0.02) and cardiac stress testing (7.8% vs 8.1%, OR = 0.96 p = 0.04), and the clinical data study indicated worse performance on blood pressure control in the intervention arm (52.8% vs 63.5%, OR = 0.72, p = 0.04), with fewer in the intervention arm achieving target range [5]. While the (unexpected) worsening in these secondary outcomes in the intervention group could represent a chance finding, there are other possible explanations. This paper deepens our understanding of the study results.

## Methods

### Qualitative study design

Our qualitative formative evaluation [23] sought to study the strategy for guideline implementation employed in the trial and to identify opportunities for improvement. Because the toolkit was distributed to family physicians, we documented experiences of family physicians with the toolkit and in managing CVD risk specifically. Qualitative methods are well-suited to understanding the lived experiences of practitioners in context [24, 25]. In designing the study we considered the possibility that there might be multiple factors influencing the uptake of the toolkit, physicians’ understanding of it, and their interests in (and motivations for) practice change. For example, features of their practice environments (e.g. workload, scope of practice), of the broader healthcare system, characteristics of physician-patient interactions and relationships, and understanding of the clinical issue itself could all influence practice behaviour and guideline uptake.

The study was comprised of two qualitative data sources: 1) written commentary from reflective feedback forms collected from the pool of 80 Ontario family physicians (40 intervention and 40 control, representing approximately 10% of the practices randomized and approached) who participated in a chart audit as part of the clinical data study of the RCT [5]; and 2) in-depth semi-structured telephone interviews with family physicians who were likely to have received the toolkit. All physician participants offered their perspectives before the trial results were released. We describe the sampling and data collection for each data source separately below.

#### 1) Written commentary from practice audit forms

**Participants providing written comments from practice audit forms:** As part of the clinical data study, a subset of 80 family physicians were recruited who were each willing to take part in a chart audit of their patients for trial outcomes, and willing to complete a reflective feedback exercise related to their practices. The chart audit occurred after the control arm had received the toolkit, that is, six months after the participants in the intervention arm would have been sent their toolkits. Half of the 80 physicians were selected from the intervention arm of the RCT (received the toolkit in the summer of 2009) and half from the control arm (received in the spring of 2010) [5, 20]. Once participants and their practices were recruited, the study team randomly selected 20 diabetic patients from each practice who were considered at “high risk for cardiovascular events” according to the CDA definition [5]. Clinical data relevant to CVD management of diabetic patients were collected via chart audit on each of these patients. Each of the physician participants in this study received a chart audit report. They were also asked to complete a reflective chart audit feedback exercise using a pre- and a post-audit form. Qualitative data (based on written text) were collected as part of this reflective chart audit feedback exercise.

**Data collection from practice audit forms:** Written commentary was captured from two paper-based forms before and after the practice audits took place. On the pre-feedback forms, participants were asked to indicate their agreement with statements pertaining to their self-reported awareness regarding: CVD and its potential complications in diabetes; whether they were aware of treatment target recommendations to reduce CVD; and whether they believed that they could achieve the recommended targets for most of their diabetic patients. Participants were also asked to reflect on the proportion of their at-risk diabetes patients who were meeting targets and if they could think of ways to better achieve CVD treatment targets for diabetes patients in their practices. Participants were provided with space to write comments and explain their answers.

On the post-feedback forms participants were asked to reflect on a new series of statements and explain their responses. They were asked to reflect on whether they thought the chart audit reports they had received were generally accurate, whether they were surprised by their audit results, and whether they were motivated to make changes in their clinical practice. On this form, they were asked what they had learned from the audit/feedback process, what they encountered as barriers to change and whether they thought this exercise was useful. Again, blank space was provided where they could comment on the chart audit process and expand upon their answers. The comments both pre- and post-feedback were used for qualitative analysis and are a focus of this paper. 65 pre-feedback forms and 62 post-feedback forms were received, for a total of 465 separate comment fields completed (129 pre and 336 post). The greater number of comments on the post-feedback form reflects the greater number of comment fields provided.

#### 2) In-depth interviews with family physicians

**Participants and challenges with recruitment:** Thirteen individual in-depth semi-structured interviews with family physicians were conducted. Our original intent had been to conduct 6 to 8 focus groups with family physicians in southern Ontario, but this had to be changed because of difficulties with recruiting and scheduling busy family physicians for this activity. Individual interviews were adopted as an alternative strategy since they were easier to coordinate with physicians’ schedules. A pragmatic approach to sampling was adopted, using a combination of purposive and snowball sampling techniques [26]. We sought to recruit physicians from the Greater Toronto Area and beyond, using our networks from the investigative team and our practice colleagues. We asked our clinical co-investigators for their assistance in identifying family physician colleagues with a range of backgrounds and experiences, as well as contacts from university-affiliated family practice departments. We also attempted recruitment at conferences/meetings frequented by family physicians. Our goal was to optimize sample heterogeneity and obtain the perspectives of those with a range of practice experiences (in keeping with qualitative methodology) [26, 27]. Obtaining a sufficient sample of interview participants proved challenging, in part because family physicians appeared to be unaware of the toolkit and were unaware of the trial (by design) [5]. Despite the challenges associated with recruiting family physicians, interview participants with a range of experiences and practice contexts were recruited. Participants ranged from recent graduates to those approaching retirement. And their interviews yielded important information about their practices, their understanding of CVD screening and awareness of the guidelines, and insights regarding the guideline implementation strategy used and the toolkit itself.

**Data collection for interviews:** Telephone interviews were conducted between July 2011 and January 2012. As mentioned above, participant recruitment proved challenging so the interviews were conducted after the trial had finished (although the results of the trial had not been released). All interviews were audio taped and transcribed. The in-depth individual interviews were based on interview guides developed by experienced qualitative researchers. Face and content validity of the guides were assessed by other team members with clinical expertise. The interview guide was structured to elicit participants’ perspectives on CVD risk assessment and management as practiced prior to the mail out of the toolkit; and regarding barriers/facilitators to the uptake of the educational tools. Participants discussed the characteristics of the tools that led to their acceptability (or lack thereof), and were invited to offer recommendations for improving guideline uptake.

### Data analysis

In keeping with qualitative methodology, interview data collection and analysis occurred in conjunction, in an iterative process [28]. For the written comments, data analysis followed data collection. We adopted an approach of qualitative description (as described by Sandelowski, 2000; 2010), which entails an inductively-derived thematic analysis[29, 30]. Interview transcripts as well as pre- and post-audit written comments were analysed for emergent themes and categories, using the technique of constant comparison within and across interviews and across data sources [28]. Data management was facilitated using NVivo software (version 9).

Techniques for ensuring analytic rigour included questioning and checking [28]. Multiple readings of the transcripts were conducted by three team members with qualitative expertise, in order to interrogate the developing coding scheme and emerging analysis [27]. Alternative explanations were explored in order to develop the most plausible and robust interpretation of the qualitative findings. Interview data were compared with the written comments from the questionnaires in order to deepen the understanding gained from both datasets.

### Ethics Statement

The study was approved by the research ethics boards of St. Michael’s Hospital and Sunnybrook Health Sciences Centre, in Toronto, Canada. All physicians in the province of Ontario were part of this pragmatic RCT. Physician participants who were part of the chart audit subgroup gave verbal consent to be part of the clinical data study. Interview participants were not part of the chart audit group and gave written consent to be interviewed.

## Results

Because of the small number of in-depth interviews, as well as the wealth of written feedback offered by physicians from the 80 practices participating in the chart audit exercise, we have synthesized the analyses of both datasets in an integrated fashion below, identifying the source dataset for each supporting quote offered. Participant IDs indicated in the manuscript text that begin with ‘I’ are for quotes from interviewees, while those beginning ‘PR’ and ‘PF’ are from the pre-audit or post-audit feedback forms respectively. Similar themes were identified in both datasets. Overall, the physician participants in our study did not perceive the toolkit to be an innovative or useful tool in terms of helping them to manage CVD risk factors and related complications from diabetes. They indicated that this was because they perceived themselves to be well aware of the guidelines prior to receiving the intervention. Moreover the dissemination and implementation strategy (mail out) was considered weak. The feedback offered by the participants can be divided into four main themes: *impressions of the toolkit*, *awareness was not the issue*, *it’s not me it’s my patients*, and *chart audit as a more effective intervention*. We consider each of these themes in detail below. We note however, that much of what follows is participants’ perceptions of their own practices; this is not to imply that we take all of their remarks/assertions at face value. Rather we use the thematic results to characterize the challenges encountered by these family physicians in managing CVD risk factors in the context of diabetes care, and to understand the shortcomings of our intervention implementation strategy.

### Physicians’ impressions of the toolkit

Feedback on the toolkit itself was directly assessed via the in-depth interviews. While participants liked some features of the toolkit (e.g. bright colours, CDA branding, succinct and organized summary of the guidelines), overall their interview accounts emphasized its redundancy, and that it contained information they *felt* they already knew. One participant commented,

I kind of looked at it and, and put it away because I thought okay, well I already know the numbers. (IP1)

Another remarked,

So if I you had to pick the two most important pages I think it would be the first page of what’s up, the second page and then the assessment tool would be the most important. Everything else is all just a reiteration of what we kind of know from med school and reading. (IP7)

The first two pages of the toolkit referred to above includes a brief summary of which patients with diabetes are at risk for CVD and rationale for screening them, which elements of the relevant clinical practice guideline they can go to for further information, as well as a treatment algorithm (e.g. which clinical findings would prompt the physician to order an ECG or stress test, etc.).

As we shall see in later sections however, while they said that they felt familiar with the information contained within the toolkit, the chart audit results and the comments from physicians on the post-audit feedback forms suggest that there is room for improvement in performance.

There were some parts of the toolkit that made a favourable impression on participants however. The laminated card was by the far the preferred item in the kit.

So the small little half page, that’s what I would really focus on because … I would assume rather that any really, really important information would probably have been distilled from (the) two larger pamphlets to this one. (IP3)

They particularly appreciated the card’s flowchart format (including a simplified algorithm for cardiovascular risk assessment) to be a handy reminder that they could see themselves using in future. Most interviewees said that this was the only component of the tool kit that they would realistically use or keep in their practice.

Reactions to the guideline summaries (4-page and 8-page booklets) included in the toolkit were mixed. Some appreciated the streamlined summary they provided:

… the actual guideline is about 200 pages, it’s probably not the most realistic thing to consistently be reading, so I do like having it in this sort of organized format and 4 or 5 pages…and from what I am quickly seeing here is it looks like a pretty good summary (IP11)

Others felt that these were still too long, and expressed a preference for the laminated card. However, a few participants felt the card itself was too wordy and would have benefited from more diagrams and less text.

The tear-off sheets for patients were not well-received by the physician participants. Most described them as “wasted paper”. Nevertheless a *few* suggested that this component could help to reinforce patient adherence to treatment:

…giving the patient something like this [gives] them some of that responsibility. So they know they are semi responsible for their type 2 diabetes (IP13).

A related sub-theme that emerged from both the interviews and the reflective feedback forms was *acceptance of guideline content*. A few of the chart audit participants expressed skepticism regarding the toolkit content, commenting that the guidelines were not based on reliable evidence. One participant commented:

blood pressure targets are often not achievable and are supported by only level 3 evidence. The evidence supporting lipid targets is also weak (PR47).

But most participants (both from interviews and chart audits) expressed an interest in adhering to the guidelines.

In general, the appearance of the toolkit materials was well-received, including its colour-scheme and branding by the CDA. “The appearance looks great… I like the colours, to be honest with you, it’s clearly CDA”(IP13). The use of logos from pharmaceutical companies got more mixed reviews, with some perceiving that it might diminish credibility of the toolkit, if only slightly: “I appreciate the fact that they contribute and without them I suppose none of this would exist. At the same time though um, I don’t know…if it lends credibility to it” (IP1). Others were less concerned, although they still felt such industry branding should be discrete.

Many of the interview participants commented that they did not recall seeing the toolkit prior to being interviewed. They saw the mail-out strategy as weak and not an effective approach to target family physicians or encourage uptake. Most said that they do not open their own mail, and even when they do, they flip quickly through what is sent to them and put many mailings in the recycling bin.

I look at them to see if there’s anything new, educational information that I think things that are new that I have not been practicing. If there is then I will read it over more. If not then honestly they all get recycled. (IP3)

The busy, high-volume nature of their clinical practices and multiple demands for their attention also acted as contextual barriers to uptake. They suggested alternative strategies to mail-out, including dissemination via interactive educational sessions with peer experts, or embedding the information within the electronic medical record (EMR).

### Awareness was not the issue–or so they thought

The majority of participants characterized themselves as being well aware of the guidelines included in the toolkit, and that they had been so well before the start of the trial. All participants said they were aware of the current practice guidelines and target numbers for key clinical indicators. Written commentary on the pre-audit forms indicated that they *perceived* their level of awareness to be high. Interview participants explained that the information contained in the toolkit was already embedded into their clinic routines and had been since the beginning of their medical careers.

In medical school we were taught that all diabetics were high risk, so technically all LDLs should be less than 2.0, they shouldn’t be smoking, their blood pressure should be 130 over 80 or less um, and so, that’s kind of what we follow. So I think the screening process is pretty similar to that. (IP7)

Interviewees commented on their screening, prevention and management routines. Tests mentioned included ECG, blood work, serum cholesterol, blood pressure assessment and management, exercise stress testing, as well as counseling regarding diet and exercise. As one physician commented, “I test their blood every 3 months with a hemoglobin A1c and a cholesterol as necessary” (IP5). Another stated,

Um, if ….you’re a diabetic they would get an ECG–if there’s ECG abnormalities, I am more likely to do an echo(cardiogram) and a stress test. (IP6).

This was echoed in the physicians’ commentary in the written feedback:

Diabetes is one of the most important causes of CVD. This is why we emphasize risk factor reduction of CVD in ALL diabetics and pre-diabetics. (PR37)

Given their sense that they had this aspect of their practice well covered, the post-audit feedback commentary suggested that their own audit results came as a surprise. Participants expressed confidence prior to the chart audit that they felt they were doing a good job managing their patients and that they expected to score highly relative to other participants on their management strategies during the trial. However, when their individual post-audit reports were received, their feedback forms indicated that participants were truly surprised that they were falling short of where they had anticipated. Comments such as “I was surprised (by the results)” and “I thought I was doing a better job of achieving targets”(PF84) were commonplace, regardless of whether the participants were in the intervention or the control group. As one participant observed,

I was surprised and disappointed by how poorly I performed on some parameters. It was surprising that 85% of patients were on statins yet only 45% at target. I would have guessed my clinical action for BP would be my worst parameter, yet I scored 100%. (PF11)

This suggests that even when they are doing well, they may not be aware of which aspects of practice they are meeting the goals.

Given that they did not perceive a need for improving their own practices prior to the RCT and their surprise at their lower-than-expected scores on the chart audit exercise, how did participants account for this disconnect? They offered a number of possible explanations, outlined under the following themes.

### It’s not me, it’s my patients

The physician participants in our study characterized the primary obstacle to achieving target outcomes (as experienced in their practices) as “[getting] patients to take medication and adhere to diet” (PR28). Put another way, they saw the limiting factor being their ability to influence their patients’ motivation and willingness to change behaviour. As one participant commented, “[we] know that there are a number of modalities available to achieve these targets if the patient is willing and compliant” (PR39).

Both the interviewees and chart audit participants emphasized that there was only so much that they could do, and that ultimately patients needed to take on at least some responsibility for their own care. As one participant wrote,

I am aware of which patients are meeting targets. The ones who are not are often non-compliant and reluctant to be treated. I don’t know of anything I can do to raise compliance in those non-compliant patients. (PR85)

Another commented,

When I diagnose someone with DM, I spend a lot of time counseling and entreating them to make every effort to send them for diabetic education. I tell them at the start how and when they need to be and try to (help) them at every opportunity. For some patients, it is difficult to get them to come in for regular follow-up and it is difficult to have the staff call them regularly…(PR40)

Participants attempted to unpack the barriers to patient behaviour change. Some cited a reluctance on the part of some patients to accept their diagnosis of diabetes (“… a lot of people deny it”–IP6). Others commented that their patients are frequently reluctant to take medications–e.g. “I think people are, are much more resistant to taking medications and much more open to investigation; they always want more blood work and less drugs” (IP3). Participants also said that some patients may experience fear, or they may feel ashamed for not being able to comply with physicians’ directives, such as failing to reach their target weight or to make prescribed lifestyle changes. Finally, patient sociodemographic factors were noted as sometimes impeding optimal management as dictated by the guidelines, and meant that they sometimes did not agree with a ‘one-size-fits-all’ approach. For example, a few participants commented that it was more difficult to reach target values in their elderly patients:

…many of my diabetics are 80 to 100 years old. Their targets cannot always be set as low as for younger patients without the risk of falls, with hypotension and hypoglycemic episodes (PF33).

Others commented that patients experiencing low socioeconomic status (SES) might have trouble adhering to physician recommendations and therefore make it difficult to reach guideline targets. Medications and healthy dietary choices can be costly, coupled with issues of access and time (time to exercise, time to find nutritious alternatives). In addition, physicians commented that persons experiencing low SES “often have multiple comorbidities as well” (IP11). This can be compounded by insufficient insurance coverage or a lack of extended health benefits.

… because in the community health centre we’re dealing with a lot of people who are working poor, don’t have health insurance, they don’t have drug insurance and they just cannot afford you know, they might be able to afford one medications, (but) when you get up to two or three…” (IP11)

The remarks about patient age and SES suggest that physicians have concerns about a lack of individualization of the existing guidelines. Evidence is important but it needs to be contextualized.

To summarize this theme, physician participants said the primary barrier to meeting target values in patients related more to patient-level factors, rather than their own unwillingness to follow the guidelines. Indeed, they indicated that they were well-aware of the guidelines, but described circumstances which made it difficult for them to reach target goals with certain patients. They perceived the primary barrier to be related to patient compliance. Identified gaps were instead self-management education and support, as well as organization of care. Interestingly, this theme contrasted with the comments of most interviewees who regarded the tear-off sheets for patients as ‘wasted paper’; this contradiction *could* be interpreted as ambivalence on the part of participants regarding notions of responsibility for adherence and speaks to it as a challenging area of practice.

### Chart audit as a more effective intervention

The chart audit component of the trial represented an interactive educational experience for physician participants. As mentioned above, the majority of the clinical data study physicians expressed surprise at their audit results: “I was surprised at the level of low target LDL-C lipids in my diabetic population” (PF2). They repeatedly expressed a renewed motivation to make changes to their practice, based on the audit results, and to be “more vigilant and aggressive with the treatment goals” (PF34). Participants commented on wanting this kind of practice feedback in future, and seemed excited by the results of the feedback. For example, it appeared that participants were surprised to learn that their diabetic patients were not necessarily being weighed at every visit, and indicated that they intended to be more aggressive in this regard. Others said that they would “focus more strongly on getting patients to target for A1c, LDL and BP” (PF11). While one participant expressed a determination to strive for optimizing patient outcomes (“I would like to see 100% of my patients at target”–PF40), still others indicated that they had “already adjusted practice to improve HbA1c goals” (PF63). These remarks are particularly salient because they indicate that glucose control in high risk patients may still be top-of-mind for most clinicians, while the recent guidelines are focused more on CVD risk management. Taken together, the emphasis on blood sugar control in their comments is suggestive that priority setting might be an issue. Busy clinicians may have trouble focusing on what they perceive to be *two* high priorities in their high risk patients. This was borne out in some of the participants’ comments, wherein they frequently referred to blood glucose management as a major priority: “I will do blood work … so like fasting sugars and …lipids and then I may stratify them with their, with other blood tests” (IP8).

Furthermore, primary care does not just entail diabetes care, but working with patients with multiple comorbidities, which presents still further challenges. As one interviewee (who did not participate in the chart audit) commented,

…but when you’re actually in these clinical encounters the chances that you will remember to do every single recommendation for diabetes, for colon cancer, for breast cancer … you’re not going to remember all of that stuff (IP3).

Despite this concern over priority setting, overall, clinical data study participants viewed the chart audit exercise as being useful, because it reinforced that they “should be more aggressive” (PF2, PF6, PF63) and highlighted where improvements could be made. Participants indicated that they would “welcome another survey to determine if I had improved” (PF20).

## Discussion

This qualitative study offers important insights as to why the pragmatic RCT evaluating the CVD toolkit demonstrated no effects, and possibly some harms [5]. The quantitative investigation revealed that the printed educational materials in the toolkit did not improve quality of care, nor did it improve cardiovascular outcomes in patients with diabetes [5]. The physician participants in this qualitative evaluation indicated that the mail-out strategy was ineffective and overly passive, with most interviewees unable to recall receiving the toolkit. Some suggested that alternative strategies could include more interactive dissemination strategies (e.g. education sessions with peer experts, or embedding the tools/prompts within the EMR). Moreover participants’ impressions of the toolkit were that it was not providing any new information, and they characterized themselves as well-aware of the practice guidelines prior to its receipt. Despite this, physicians’ reactions to their individual post-audit feedback were typically ones of surprise, as they had felt that they were “doing a better job” of meeting target values for their diabetic patients and providing recommended assessments than they actually were. Participants’ offered a number of explanations for why they might have difficulty meeting the recommended targets, with the most common being patient-related factors. Participants said that patient compliance (or lack thereof) with treatment recommendations was the biggest obstacle to achieving target outcomes.

It is important to note that throughout this paper, we have been reporting participants’ impressions, opinions and perceptions–these are not meant to be accepted at face value, but rather require careful interpretation. Taken together, their accounts suggest some important challenges inherent in managing CVD risk in their diabetic patients. Participants recounted that it was difficult to achieve treatment to target*s* (emphasis plural–targets for CVD management *and* blood sugar control). Recurrent references to HbA1c in their accounts suggest that participants may be struggling to prioritize CVD management (as recommended in the guidelines) over blood sugar control, and that they are getting caught between (or distracted by) what they see as ‘competing’ priorities [31]. We speculated in our prior quantitative paper that the mail out of the toolkit followed closely on the release of the 2008 guidelines, which may have shifted physicians’ focus to other aspects of diabetes care and away from CVD management. A recent study by Ivers and colleagues (2014) echoes our findings, in that family physicians in that study noted challenges in priority setting in their busy practices [31].

The findings from the qualitative investigation help to clarify some of the quantitative findings reported previously [5]. The statistical analysis comparing the treatment and control arms revealed no difference between the two groups in terms of physicians’ prescribing of statins and ACE-inhibitors (> 85% for *both* arms, which may indicate a ceiling effect) [5]. While the differences in ordering ECG and cardiac stress tests reached statistical significance between the intervention and control groups (and were in fact in the *wrong* (unintended) direction), these differences were not necessarily clinically important (38.8% intervention, 40.2% control for ECG testing, p = 0.02, 7.8% intervention, 8.1% control for cardiac stress testing, p = 0.04) [5]. The qualitative findings confirm that participants felt they were well-aware of the guidelines and they said that they had already implemented the treatment recommendations into their daily practice. The high ratings they gave themselves on CVD management in their pre-audit feedback suggest a self-perceived high level of awareness before the audit began. The fact that they expressed surprise at their scores on the chart audit exercise post-audit (regardless of group) suggests that they had *assumed* that they were doing what they needed to do for their diabetic patients. Thus they may not have made any efforts to change their individual practices based on the toolkit’s recommendations until after the audit was over, since they were unaware of this until after receiving the audit feedback at study completion. This was true for both intervention and control group participants. Our study participants had *unperceived needs*, and intervention design should include a mechanism for identifying such needs first and foremost (bringing them to the physician’s attention) in order to inform the design and implementation of any intervention [14]. They did not know what they did not know, which meant that the toolkit was unable to inform practice improvement. It is hard to improve something when the need for improvement is unrecognized. It was not that our participants were unaware of the guidelines per se [32, 33] rather they were unaware of the shortcomings in their own performance. The fact that they perceived themselves to be adhering to the guidelines better than they actually were suggest that although they were aware of the need for cardiovascular intervention, they had apparently not prioritized it; even so, they were expecting themselves to have reached a very high standard of performance. Referring to the Pathman-PRECEED model of knowledge translation (outlined by Davis et al, 2003) [33], we could infer that the intervention employed in our trial was targeted at the ‘awareness’ end of the spectrum. While general awareness and agreement were not at issue for our participants, what was at issue was the prioritization of that problem (CVD risk); and thus the decision to divert scarce attentional and time resources to adherence to the guidelines (performance) may be two key issues here [33].

What might an intervention look like to address such unperceived needs? Perhaps one of the most telling findings from the qualitative investigation was that the chart audit exercise used to assess physician performance during the trial represents a more effective tool for increasing physician awareness of (own) practice shortcomings and at motivating them to make improvements than the study intervention itself. This was an unanticipated consequence of the trial and suggests that this kind of feedback exercise would be a far more effective intervention, as it challenges physicians’ assumptions about their own performance [30]. Certainly the knowledge translation literature is replete with evidence that passive knowledge translation strategies are less effective than more active forms [14]. One of the reasons the chart audit exercise was perceived to be more motivating for practice change by our physician participants was its being embedded within their own practices, offering individualized practitioner feedback, based on their own patients’ data [33]. We also included peer comparison for benchmarking, which audit interventions do not often include. Our findings suggest that participants overestimated their own CVD management practices, and that the feedback challenged their assumptions [14]. However, as Grol and Grimshaw (2003) caution, audit and feedback alone–while effective for targeting test ordering and prevention–will not necessarily lead to sustained effects [14]. Indeed the evidence for audit and feedback is problematic. Ivers and colleagues (2013) found no effect in improving primary care performance in diabetes in a trial which added goal setting and action planning to audit and feedback versus audit and feedback alone [34]; and a related qualitative evaluation revealed that competing priorities and a perceived discordance between meeting population-based quality targets and delivering patient-centred care [31]. It is likely that a multifaceted approach, using educational materials, audit and feedback, as well as reminders may be more likely to result in behaviour change [14, 33]. The physicians in our qualitative study identified multiple other avenues for improving guideline uptake in primary care, where there are many other issues competing for their attention. Embedding prompts into systems of care (e.g. via EMR) and leveraging peer experts during face-to-face meetings were some additional suggestions they offered. The Pathman-PRECEED model advocates that KT interventions should be targeted to different ‘stages of change’ [33]. The qualitative findings outlined here suggest that participants were at the “adoption”/“adherence” phases of that model, while the guideline dissemination strategy was targeted at the level of “awareness.” [33] Participants told us that they were well aware of, and agreed with, the guidelines in most instances. As such, the distribution of the printed material served as ‘preaching to the converted’. In contrast, the chart audit exercise was more appropriately targeted to enabling improved performance [33].

The physicians in our study explained difficulties in meeting targets with their patients as being driven by patient-level characteristics rather than practice-level gaps. They emphasized that a certain segment of their patients will be non-adherent to treatment recommendations. They also emphasized that patients ultimately need to take on some responsibility for managing their disease and they need to be motivated and willing to engage in behaviour change. While this may in part be true, it is important to emphasize that health care providers have an important role to play in supporting and coaching patients in self-management, which includes improving adherence. Participants in our study also talked about the particular challenges of achieving targets in some patient groups, namely elderly patients with multiple comorbidities, and those experiencing low SES. As Boyd and colleagues (2005) point out, relatively few clinical practice guidelines address their application in more complex patient populations (although updated 2013 CDA guidelines do address this), which inhibits their real-world application [35]. The underlying theory of change [22] in our RCT was that the intervention would influence practitioner behaviour, which would translate into improved patient outcomes. However, if the intervention is not resonating with practitioners, there are unlikely to be any detectable positive effects on patient outcomes. Furthermore, the toolkit was developed without any specific quality improvement/educational theory^[^^36^^]^ to guide content and delivery, which might have improved its design, implementation and uptake by end-users [5].

### Limitations

It was extraordinarily difficult to recruit family physicians to participate in the interview component of the study. This is not uncommon in studies with this population [37]. However, we consider the difficulty with recruitment in this instance to be a finding in itself, in that it could be interpreted as reflecting a profound *disinterest* in the toolkit on the part of family physicians, and the ineffective nature of the mail-out strategy for disseminating the guidelines. As the results outlined above attest, the physicians participating in the RCT and the in-depth interviews stated that they felt that they already knew the guidelines well and that they did not perceive the toolkit to offer additional information of which they were unaware. On the other hand, it might also be that busy family physicians simply do not have time to participate. While the written comments indicate that these participants were surprised by what they did not know (and by their own performance during the audit exercise), prior to their experiences of audit they felt that they were performing at a high standard in terms of CVD management with their diabetes patients.

### Concluding remarks

The study provides important evaluation data concerning the implementation and results of this specific pragmatic cluster RCT using an educational toolkit as a KT intervention. The toolkit was found to not have any positive effects on patient outcomes, and practice-level variables revealed that there was little difference between the intervention and control arms of the study. The qualitative findings outlined in this paper indicate that physicians did not perceive the implementation strategy or the content of the toolkit itself to be particularly informative to their management of CVD risk among their own diabetic patients. They indicated that they were well-acquainted with the practice guidelines prior to receiving the toolkit. However, this study also provides useful formative evidence in the design of any intervention aimed at improving the performance of physicians in relation to cardiovascular care of their patients with diabetes. The results of the qualitative study also demonstrate that physicians had unperceived needs relative to CVD management, of which they were not aware, and that the chart audit served to identify shortcomings in their practice of which they had been hitherto unaware. The findings also indicate that new methods of intervention development and implementation should be explored. Diabetes is a highly prevalent condition worldwide and appropriate CVD management is critical to decreasing the morbidity and mortality associated with it. Interventions that optimize CVD management by family practitioners are urgently needed.
